# High prevalence of chronic kidney disease and its related risk factors in rural areas of Northeast Thailand

**DOI:** 10.1038/s41598-022-22538-w

**Published:** 2022-10-28

**Authors:** Ubon Cha’on, Patcharaporn Tippayawat, Nattaya Sae-ung, Porntip Pinlaor, Wichien Sirithanaphol, Ampornpan Theeranut, Kriang Tungsanga, Prathana Chowchuen, Amod Sharma, Supakit Boonlakron, Sirirat Anutrakulchai

**Affiliations:** 1 Department of Biochemistry, Faculty of Medicine, Khon Kaen University, Khon Kaen, Thailand; 2 Chronic Kidney Disease Prevention in the Northeast of Thailand (CKDNET) Project, Khon Kaen University, Khon Kaen, Thailand; 3 Department of Medical Technology, Faculty of Associated Medical Sciences, Khon Kaen University, Khon Kaen, Thailand; 4 Department of Surgery, Faculty of Medicine, Khon Kaen University, Khon Kaen, Thailand; 5 Faculty of Nursing, Khon Kaen University, Khon Kaen, Thailand; 6 Department of Medicine, Faculty of Medicine, King Chulalongkorn Memorial Hospital, Chulalongkorn University, Bangkok, Thailand; 7 Bhumirajanagarindra Kidney Institute, Bangkok, Thailand; 8 Department of Radiology, Faculty of Medicine, Khon Kaen University, Khon Kaen, Thailand; 9 Institute of Dermatology, Department of Medical Services, Ministry of Public Health, Bangkok, Thailand; 10 Department of Internal Medicine, Faculty of Medicine, Khon Kaen University, Khon Kaen, Thailand; 11 Division of Nephrology, Department of Internal Medicine, Faculty of Medicine, Khon Kaen University, Khon Kaen, 40002 Thailand

**Keywords:** Diseases, Health care, Nephrology, Risk factors

## Abstract

In Thailand, chronic kidney disease (CKD) screening was reported in 2009 with an overall prevalence of 17.5% and the highest at 22.2% in the northeastern region. This study aimed to find out CKD prevalence of the Kidney Disease Improving Global Outcomes criteria and their related risk factors in the rural community. A population-based study was conducted in the rural sub-districts of northeastern Thailand. Data of socio-demographic status, lifestyle, underlying diseases, blood pressure, and body mass index were recorded. Blood and urine analysis was conducted along with ultrasonography of kidneys. Specimen collection and analyses were repeated after 3 months, and the factors associated with CKD were studied by logistic regression analysis. A total of 2205 participants with a mean age of 57.8 ± 11.7 years and female predominance (66.7%) completed the study. The prevalence of CKD was 26.8%, i.e., stages 1 (7.3%); stage 2 (9.0%); stage 3a (6.0%); stage 3b (2.8%); stage 4 (1.4%); and stage 5 (0.3%). Hypertension, diabetes mellitus, and renal stones were the major underlying diseases. Only 3.5% of the participants were aware of having CKD. An increase in age, male, unemployment, current smoking, diabetes, hypertension, underweight, anemia, hyperuricemia, and leukocytosis were significantly associated factors with the disease. The study revealed that CKD has developed as a significant public health problem in rural northeastern Thailand and one out of every four people has CKD. Therefore, early interventions are essential for the proper management and prevention of CKD.

## Introduction

Chronic kidney disease (CKD) is causing a huge burden in terms of occurrence, treatment cost, morbidity, and mortality throughout the world each year^1,2^. Global estimated death and disability-adjusted life years from the disease in 2017 were 1.2 million and 35.8 million^3^. Diabetes mellitus (DM) and hypertension (HT) are the main causes of CKD^4,5^. Moreover, the association of factors such as race, sex, age, diet, socioeconomic status, family history, smoking, nephrotoxin, and others with CKD has made the situation more challenging^6^. A better knowledge of epidemiology is, therefore, inevitable to discourse on its multi-dimensional effects, especially in low and middle-income countries including Thailand.

Over the past two decades, epidemiological studies have revealed a soaring national prevalence of CKD ranging from 4.6 to 17.5% in Thai populations, more severely affecting the inhabitants of the rural and low economic northeastern region^7–12^. The observed discrepancy in the prevalence was due to CKD diagnostic criteria, estimated glomerular filtration rate (eGFR) equation, study design, and population. For example, a Thai Screening and Early Evaluation of Kidney Disease (SEEK) study reported 17.5% CKD occurrence among national adult populations with 22.2% prevalence in Northeast Thailand using criteria of reduced eGFR calculated by the modification of diet in renal disease (MDRD) formula, hematuria, and albuminuria^10^. On the other hand, a recently published study revealed a lower nationwide CKD frequency of 8.7% based on the eGFR from the Chronic Kidney Disease–Epidemiology Collaboration (CKD-EPI) equation and proteinuria, however, the highest prevalence of CKD was also observed among Northeastern adults^7^. As in the majority of population-based CKD screening, these findings were based on the measurement of one-time eGFR, hematuria, and albuminuria or proteinuria. The reports on CKD burden containing additional diagnosis criteria such as kidney ultrasonography as well as the confirmation of reduced eGFR and other kidney function markers after a subsequent period are scarce. Therefore, this study was designed to investigate CKD prevalence among rural northeastern Thai populations using the recent Kidney Disease Improving Global Outcomes (KDIGO) criteria including detection of chronic renal structural abnormalities by ultrasound, and to enumerate predisposing factors as some risk factors that could be modified and may prevent or retard the progression of CKD to end-stage kidney disease (ESKD).

## Results

### Characteristics of all participants

A total of 2789 participants from 24 villages participated in the study and 2205 completed the entire screening procedure, accounting for a 79% response rate. About 19% of the participants dropped out between the interview and the first round of sample collection. A comparison between the total participants, those who completed, and dropped out of the study is shown in Table S1 in which the distribution of proportions of age, sex, education, occupation, and income between the total participants and the 2205 participants completing follow-up were similar, however, the significant differences between the participants completing follow-up and the 584 drop-out participants were revealed in such parameters that the dropped subjects were younger (53.2 vs. 57.8 years), more males (42.1% vs. 33.3%), less in farming occupation (42.8% vs. 63.5%), and had more highly educated degree and income. An average age of the final enrolled participants was 57.8 ± 11.7 years with female predominance (66.7%). A majority of the participants were educated up to primary school (79.0%), agriculture was their main occupation (63.5%), and most (90.6%) had monthly incomes of less than 10,000 Baht. The participants were underweight (4.9%), normal weight (26.6%), overweight (18.7%) with class I obesity (37.1%), and class II obesity (12.7%). The prevalence of CKD (26.8%), DM (21.0%), and HT (31.3% in which 41.7% of them had accompanying DM) was rather high. Additionally, the proportions of hypercholesterolemia (45.5%), hyperuricemia (21.1%), and anemia (32.0%) were also unexpectedly excessive.

Usage of non-steroidal anti-inflammatory drugs (NSAIDs) and analgesic drugs was common (39.0%), and 46.9% of these subjects were currently taking some of the drugs. Nearly 70% of taken medication was self-accessed and illegally obtained from peddlers or at grocery shops in the villages. The amount of salt intake was two-fold the WHO recommendation. The current use of agricultural herbicides was 28.2%. The participants with current smoking and drinking habits were 12.2% and 25.0%.

### Awareness of CKD and related diseases

Although CKD and its related diseases were discovered as a large burden in this rural region, awareness of these diseases was quite low. Only 21 of 592 CKD cases (3.5%) had perceived their renal dysfunction and structural abnormality. In other words, 96.5% of them were unaware of their CKD. Considerable percentages of unrecognized HT (23.5%), DM (22.7%), and renal stones (69.1%) were discovered.

### Comparisons between the CKD- and non-CKD groups

Baseline characteristics of the participants are described in Table 1, which shows that 592 cases (26.8%) were identified to have CKD. Education, occupation, and monthly income were statistically different between the participants with and without CKD. A higher percentage of participants with primary school, unemployed status, and less than 10,000 Baht per month income was revealed in the CKD group. The participants with CKD were older and male predominance, had greater proportions of;—underweight, DM, HT, renal stones, gout, cardiovascular disease (CVD) and higher total WBC count, uric acid levels and lower hemoglobin and LDL cholesterol levels compared to non-CKD participants.

**Table 1:** Baseline characteristics of CKD and non-CKD patients.

Variables	CKD (n = 592)	Non-CKD (n = 1,613)	*P* value
Age, years (mean ± SD)	64.0 ± 11.0	55.5 ± 11.1	< 0.001
Sex: Male (n, %)	234 (39.5)	501 (31.1)	< 0.001
Weight, kg (mean ± SD)	60.4 ± 11.9	61.7 ± 11.0	0.014
Height, cm (mean ± SD)	155.6 ± 7.52	156.4 ± 7.34	0.016
Body mass index, kg/m^2^ (mean ± SD)	24.9 ± 4.4	25.2 ± 4.3	0.123
UW/NW/OW/Obesity, (%)	7.3/26.0/18.1/48.6	4.1/26.8/18.9/50.2	0.047
Education, n (%)			< 0.001
Did not study	4 (0.6)	8 (0.5)	
Primary school	515 (87.0)	1,226 (76.0)	
Middle school	27 (4.6)	148 (9.2)	
High school/vocational	35 (5.9)	157 (9.7)	
Diploma/high vocational	5 (0.8)	31 (1.9)	
Bachelor’s degree	6 (1.0)	38 (2.3)	
Postgraduate	0 (0.0)	5 (0.3)	
Occupation, n (%)			< 0.001
Farmer	347 (58.6)	1,054 (65.3)	
Trader	20 (3.4)	69 (4.3)	
Jobs in factories, companies	3 (0.5)	38 (2.4)	
Government/enterprise	6 (1.0)	27 (1.7)	
Student	0 (0.0)	8 (0.5)	
Other	49 (8.3)	199 (12.3)	
Unemployed	167 (28.2)	218 (13.5)	
Monthly income (Baht), n (%)			0.001
< 10,000	556 (93.9)	1,442 (89.4)	
> 10,000	36 (6.1)	171 (10.6)	
Underlying diseases HT (overall), n (%)	307 (51.9)	383 (23.7)	< 0.001
HT (without DM), n (%)	147 (24.8)	255 (15.8)	< 0.001
HT (with DM), n (%)	160 (27.0)	128 (7.9)	< 0.001
DM, n (%)	212 (35.8)	250 (15.5)	< 0.001
Renal stone, n (%)	128 (21.6)	21 (1.3)	< 0.001
Gout, n (%)	24 (4.1)	31 (1.9)	0.004
CVD, n (%)	15 (2.5)	16 (1.0)	0.006
SLE, n (%)	4 (0.7)	12 (0.7)	1.000
Serum creatinine (mg/dL) (mean ± SD)	1.1 ± 0.7	0.8 ± 0.2	< 0.001
eGFR, ml/min/1.73 m^2^ (mean ± SD)	70.4 ± 25.4	91.9 ± 14.5	< 0.001
UACR, mg/g [median (IQR)]	26.7 (6.5–109.6)	5.8 (3.6–10.4)	< 0.001
Hemoglobin, g/dL (mean ± SD)	12.3 ± 1.7	12.9 ± 1.3	< 0.001
Anemia, n (%)	278 (47.0)	426 (26.4)	< 0.001
Female	184/358 (51.4)	306/1,112 (27.5)	< 0.001
Male	94/234 (40.2)	120/501 (24.0)	< 0.001
Leukocytosis, n (%)	90 (15.2)	157 (9.7)	< 0.001
Serum WBC, cells/ml (mean ± SD)	7,987 ± 2,189	7,369 ± 1,886	< 0.001
Hyperuricemia, n (%)	230 (38.9)	236 (14.6)	< 0.001
Uric acid, mg/dL (mean ± SD)	6.1 ± 1.7	5.2 ± 1.3	< 0.001
Hyperlipidemia, n (%)	226 (38.2)	778 (48.2)	< 0.001
LDL cholesterol, mg/dL (mean ± SD)	122.0 ± 39.6	129.9 ± 37.9	< 0.001

*CKD* chronic kidney disease; *UW* underweight; *NW* normal weight; *OW* overweight; *HT* hypertension; *DM* diabetes mellitus; *CVD* cardiovascular disease; *SLE* systemic lupus erythematosus; *eGFR* estimated glomerular filtration rate; *UACR* urine albumin creatinine ratio; *WBC* white blood cell; *LDL* low-density lipoprotein; *SD* standard deviation.

Regarding the association of CKD with behaviors, smoking habits, NSAID and analgesic use, and alcohol consumption were significantly different between the CKD and non-CKD groups. Daily water intake was significantly lower in the participants with CKD than without CKD. No significant differences were found for the variables such as usage of herbicide, herbs, and daily salt intake between the two groups (Table 2).

**Table 2:** Possible behaviors related to chronic kidney disease.

Variables	CKD (n = 592)	Non-CKD (n = 1613)	*P* value
Smoking habits			0.011
No	427 (72.1)	1263 (78.3)	
Quit	78 (13.2)	168 (10.4)	
Till now	87 (14.7)	182 (11.3)	
Total DOC and Total NSAID use, n (%)			0.020
Never used	389 (65.7)	957 (59.3)	
Used to take	112 (18.9)	344 (21.3)	
Still taking	91 (15.4)	312 (19.3)	
Herbal use, n (%)	99 (16.7)	296 (18.4)	0.377
Drinking water, mL/day (mean ± SD)	1933 ± 932	2054 ± 964	0.009
Salt intake, g/day (mean ± SD)	4.1 ± 4.2	4.2 ± 4.6	0.709
Fish sauce intake, ml/day (mean ± SD)	9.5 ± 7.0	9.2 ± 8.0	0.425
MSG intake, g/day (mean ± SD) Fermented fish sauce intake, ml/day (mean ± SD)	4.8 ± 9.7 9.5 ± 10.5	5.0 ± 5.4 8.6 ± 9.5	0.646 0.070
Alcohol consumption, n (%)			0.010
Do not drink	387 (65.4)	1028 (63.7)	
Used to drink	79 (13.3)	160 (9.9)	
Continued drinking	126 (21.3)	425 (26.4)	
Exercise, n (%)	327 (55.2)	905 (56.1)	0.743
Frequency, times/week [median (IQR)]	6 (3–7)	5 (3–7)	0.361
Use of herbicide			0.638
Never	381 (64.4)	990 (61.4)	
Have used	50 (8.5)	163 (10.1)	
Still using	161 (27.2)	460 (28.5)	

*CKD* chronic kidney disease; *DOC* drug over the counter; *NSAIDs* non-steroidal anti-inflammatory drugs; *MSG* monosodium glutamate; *IQR* interquartile range; *SD* standard deviation.

### Characteristics of CKD in the rural community

The distribution of eGFR and CKD cases according to sex and age group are shown in Table S2 and Figs. S1, S2. About one third of age group 60–69 years and a half of age ≥ 70 years participants had CKD and males revealed higher CKD prevalence than females for all age groups. The causes of CKD were identified in 454 of 592 cases, when DM, HT, and renal stones were the top three etiologies; 23.3% of CKD subjects (6.3% of all participants in the study) were of unknown causes. Table S3 summarizes the total cases investigated in rounds 1 and 2 of examinations and numbers of CKD cases defined by various criteria of KDIGO. Proportions of CKD according to criteria of persistent eGFR < 60 mL/min/1.73 m^2^ were 39.2%, albuminuria 49.0%, hematuria 17.1%, and abnormal renal ultrasound 56.3% in which parenchymatous changes and renal stones were the main findings (Table S4). Notably, 123 cases accounting for 20.8% of those CKD subjects, or 5.6% of total cases, were classified as CKD by abnormal ultrasound findings alone without any other CKD criteria.

**Figure 1:**
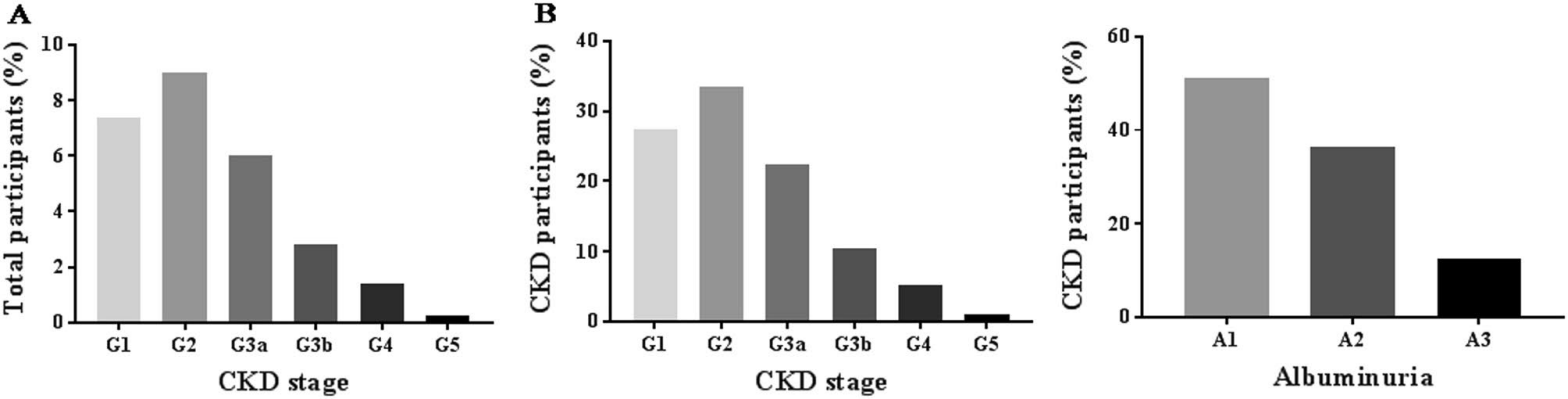
(**A**) Prevalence of CKD classified as GFR category (G1-G5) in total participants, and (**B**) Distribution of CKD stages (G1-G5) and albuminuria category (A1-A3) in the CKD population. Note that the majority of participants were in the early stages of CKD.

As shown in Fig. 1, the prevalence of each CKD stage according to eGFR levels assessed by CKD-EPI formula were G1; 7.3% (162 cases), G2; 9.0% (198 cases), G3a; 6.0% (133 cases), G3b; 2.8% (62 cases), G4; 1.4% (31cases), and G5; 0.3% (6 cases). Proportions of CKD subjects classified by albuminuria category were A1; 13.7% (302 cases), A2; 9.8% (215 cases) and A3; 3.4% (75 cases). Additionally, proportions of albuminuria categories in each CKD stage are shown in Fig. S3 and distributions of CKD cases in each GFR and albuminuria categories in which CKD was defined by hematuria and ultrasound criteria are illustrated in Fig. S4.

Prevalence of CKD increased to 27.8% with stages 1 (122 cases, 5.5%); stage 2 (224 cases, 10.2%); stage 3a (139, 6.3%); stage 3b (93, 4.2%); stage 4 (30, 1.4%); and stage 5 (6 cases, 0.3%) if CKD was defined and classified by eGFR levels assessed with the MDRD formula. A comparison of CKD prevalence in the population according to eGFR levels calculated by CKD-EPI, MDRD and the reported Thai Eqs. ^13^ is shown in Table S5.

### Factors associated with CKD

The univariate logistic regression analysis of the risk factors associated with CKD is shown in Table 3. Notably, the odds of having CKD were higher in combined HT and DM as compared to either DM or HT. Multivariate analyses to represent the adjusted ORs of risks were applied for two models namely Model 1 and Model 2. The first model used continuous variables:—SBP, BMI, plasma glucose, hemoglobin, serum uric acid, WBC count, and LDL-cholesterol, whereas the second model used categorical variables, i.e., presence or absence of being underweight, DM, hypertension, anemia, hyperuricemia, leukocytosis, and hyperlipidemia in the analyses. The variables having a significant positive association in both the models were age (OR 1.05 for every 1-year increase), DM (OR 2.18 for presence and OR 1.008 for every 1 mg/dL FPG increase), HT (OR 1.80 for appearance and OR 1.009 for every 1 mmHg SBP increase), anemia (OR 1.65 for existence and OR 0.79 for every 1 g/dL hemoglobin increase), leukocytosis (OR 1.40 for presence and OR 1.14 for every 1000-cells/mL WBC increase), and hyperuricemia (OR 2.68 for presence and OR 1.42 for every 1 mg/dL uric acid increase). Furthermore, unemployed status (OR 1.38), current smoking (OR 1.56 compared to no smoking), had significant positive associations with CKD in Model 1, and male gender (OR 1.73) and underweight (OR 1.64) in Model 2. The analysis of CKD risk factors excluding anemia and hemoglobin as variables (Table S6), and the factors associated with CKD stage 3–5 (with and without anemia and hemoglobin as variables) in the models are shown in Table S7 and Table S8. The prominent risk factors related with CKD G3-5 were age, male sex, and presence of;—DM, HT, hyperuricemia, and anemia.

**Table 3:** Factors associated with chronic kidney disease.

Factors	Crude OR (95% CI)	P value	Model 1		Model 2	
			Adjusted OR	*P* value	Adjusted OR	*P* value
Age (every 1- year increase)	1.07 (1.06–1.09)	< 0.001	1.05 (1.03–1.06)	< 0.001	1.04 (1.03–1.06)	< 0.001
Male	1.45 (1.19–1.76)	< 0.001	1.26 (0.86–1.84)	0.241	1.71 (1.20–2.45)	0.003
Low monthly income (< 10,000 Baht)	1.83 (1.26–2.66)	0.001	0.89 (0.58–1.38)	0.612	0.88 (0.57–1.36)	0.575
Less education (none plus primary school)	2.19 (1.67–2.86)	< 0.001	0.96 (0.69–1.34)	0.804	0.98 (0.70–1.37)	0.919
Unemployed status	2.51 (2.00–3.16)	< 0.001	1.38 (1.03–1.85)	0.029	1.33 (0.999–1.78)	0.051
Smoking habits		0.013				
no	1		1		1	
quit	1.38 (1.03–1.85)	0.030	0.86 (0.55–1.36)	0.523	0.80 (0.51–1.26)	0.331
continues now	1.40 (1.06–1.85)	0.019	1.58 (1.02–2.44)	0.040	1.45 (0.95–2.23)	0.087
NSAID use		0.019				
Never used	1		1		1	
Used to take	0.80 (0.63–1.02)	0.074	0.95 (0.71–1.27)	0.744	0.89 (0.67–1.18)	0.418
Still taking	0.72 (0.55–0.93)	0.013	0.80 (0.59–1.08)	0.141	0.80 (0.59–1.08)	0.143
Alcohol consumption		0.010				
Do not drink	1		1		1	
Used to drink	1.31 (0.98–1.76)	0.069	0.85 (0.56–1.30)	0.463	0.82 (0.54–1.25)	0.355
Continuous drinking	0.79 (0.63–0.99)	0.044	0.78 (0.57–1.07)	0.123	0.78 (0.57–1.08)	0.133
Drinking water (every increase of 500 ml a day)	0.93 (0.86–0.98)	0.009	0.99 (0.93–1.05)	0.644	0.98 (0.92–1.04)	0.510
Underweight (BMI < 18.5 kg/m^2^)	1.85 (1.24–2.76)	0.003			1.64 (1.03–2.63)	0.038
BMI (every 1 kg/m^2^ increase)	0.98 (0.96–1.005)	0.123	0.99 (0.96–1.02)	0.450		
DM	3.04 (2.45–3.77)	< 0.001			2.20 (1.69–2.86)	< 0.001
Fasting plasma glucose (every 1-mg/dL increase)	1.007 (1.006–1.009)	< 0.001	1.008 (1.005–1.01)	< 0.001		
HT without DM	2.49 (1.96–3.16)	< 0.001			1.80 (1.42–2.29)	< 0.001
HT with DM	5.39 (4.14–7.04)	< 0.001				
SBP (every 1-mmHg increase)	1.024 (1.019–1.030)	< 0.001	1.010 (1.003–1.016)	0.004		
Anemia	2.46 (2.02–2.99)	< 0.001			1.64 (1.30–2.07)	< 0.001
Hemoglobin (every 1-g/dL increase)	0.78 (0.73–0.83)	< 0.001	0.79 (0.73–0.87)	< 0.001		
Hyperuricemia	3.71 (2.99–4.60)	< 0.001			2.69 (2.10–3.44)	< 0.001
Serum uric acid (every 1-mg/dL increase)	1.52 (1.42–1.63)	< 0.001	1.42 (1.30–1.54)	< 0.001		
Leukocytosis	1.66 (1.26–2.20)	< 0.001			1.40 (1.001–1.96)	0.049
WBC count (every 1000-cells/mL increase)	1.16 (1.11–1.22)	< 0.001	1.14 (1.08–1.21)	< 0.001		
Hyperlipidemia	0.66 (0.55–0.80)	< 0.001			0.81 (0.65–1.01)	0.061
LDL-cholesterol (every 1-mg/dL increase)	0.994 (0.992–0.997)	< 0.001	0.998 (0.995–1.000)	0.097		

Model 1 used continuous variables of SBP, BMI, plasma glucose, hemoglobin, serum uric acid, serum white blood cells, and LDL cholesterol. Model 2 used categorical variables as presence of underweight, DM, HT, anemia, hyperuricemia, leukocytosis, and hyperlipidemia.

*NSAID* non-steroidal anti-inflammatory drug; *BMI* body mass index; *DM* diabetes mellitus; *HT* hypertension; *SBP* systolic blood pressure; *WBC* white blood cell; *LDL* low-density lipoprotein; *OR* odds ratio; *CI* confidence interval.

## Discussion

The current study is one of few population-based studies and the first in northeastern Thailand applying the KDIGO criteria to screen for CKD. We confirmed albuminuria, hematuria, and reduced eGFR after subsequent time, and performed ultrasonography of kidneys to obtain precise prevalence and risk factors of CKD in the study population. The study found that 26.8% of a representative cross-section population in study regions had CKD and the major etiologies were DM, HT, and renal stones. Moreover, age, male sex, unemployment, smoking, underweight, anemia, hyperuricemia, and leukocytosis were the significant risk factors for the CKD.

Although this CKD prevalence was unable to be directly compared with the earlier CKD screening studies in the Thai population^7,10^ because of the differences of study design, enrolled population, standard criteria used to define CKD along with the different equations for eGFR, the observed frequency of CKD in the villagers who really lived in the rural regions was very high and one explanation might be the older participants in this study (57.8 years vs. 45.2 years in Thai SEEK^10^ and 47 years in Aekplakorn, et al. study^7^). A comparison with global data in similar age subgroups and GFR values evaluated by the CKD-EPI Eq. ^14^, however, discovered that the CKD prevalence was quite high in that 17.9% of the participants were identified with CKD based on eGFR and/or microalbuminuria but the number reached 19.2% when the criterion of hematuria was applied in the study. About one-fifth of the CKD cases were identified by ultrasound alone in which renal stones and parenchymatous changes were the main findings (Table S4). Although uncommonly performed in an epidemiological study and its operator dependent, ultrasound is effective in early detection of CKD among the general population^15^.

The other fact that strengthens these results is proving the chronicity of the initially decreased eGFR (< 60 ml/min/1.73 m^2^). It was discovered that overdiagnosis of CKD was as low as 1.6% on subsequent analysis of eGFR after 3 months (Table S3). Contrarily, the discrepancies between the initial and repeated prevalence of albuminuria (4.7%) and hematuria (5.8%) were rather high (Table S3). The majority of CKD participants were identified in the early phase (stage 1–3). This is crucial especially when the progression of early-stage CKD to ESKD can be slowed down by avoiding the associated risk factors and proper care and management. Consequently, the analysis of risk factors was also attempted in the study.

One of the significant risk factors associated with CKD in the study population was age. The prevalence of CKD was substantially increased in the age groups of 60–69 and ≥ 70 years as in previous Thai and global reports^7,10,14,16^. Older age is known as a vulnerable risk for renal injury, however, this also means they have aging kidneys which may be not kidney damage and amending the CKD definition to include age specific thresholds for GFR was suggested^16–19^. In addition, although CKD prevalence is higher in women than men in most geographical regions, but variations exist around the globe as in the current study^17^. This could possibly be linked to an unhealthier lifestyle and a higher chance to contact agents related to CKD from an agricultural environment, particularly among men in this study. Another positively associated factor with CKD in the population was underweight, which may be due to the loss of muscle mass and function (sarcopenia) as reported in both non-dialysis and dialysis CKD patients but more in the latter 3 stages and aged individuals^20,21^. Supporting these current findings, underweight Chinese subjects were revealed to have a high risk for kidney failure^22^.

Current smokers had a risk for CKD compared with non-smokers as reported in previous studies^23,24^. Proposed mechanisms of renal damage by smoking were endothelial cell injury, mesangial proliferation, insulin resistance, and increases of oxidative stress and advanced glycation end products^25^. As expected, CKD was significantly associated with its traditional risk factors i.e., HT, DM, renal stones, and gout in the study population. These factors are the major challenges for CKD prevention and control in the region, especially when a swift increase in the incidence of HT and DM in Thailand had been witnessed in the past 2–3 decades^26^. This study also discovered a sizable number of unrecognized DM, HT and renal stones in rural communities. Likewise, a report has shown that more than 80% of people in Thailand are unaware of having DM, and HT^27^. Therefore, policies to screen and control DM and HT and nephrolithiasis are vital in CKD prevention.

In the present study, the magnitudes and severity of anemia were higher than an expectation from CKD complications. Although anemia is a well-known complication of CKD, some previous studies have stated that anemia by itself may be a risk factor or co-incident with CKD from other causes^28,29^. The current study revealed that hyperuricemia is associated with CKD which previous postulated mechanisms of renal injury were augmentation of systemic hypertension, afferent arteriopathy and activation of oxidative stress^30^.

**Figure 2:**
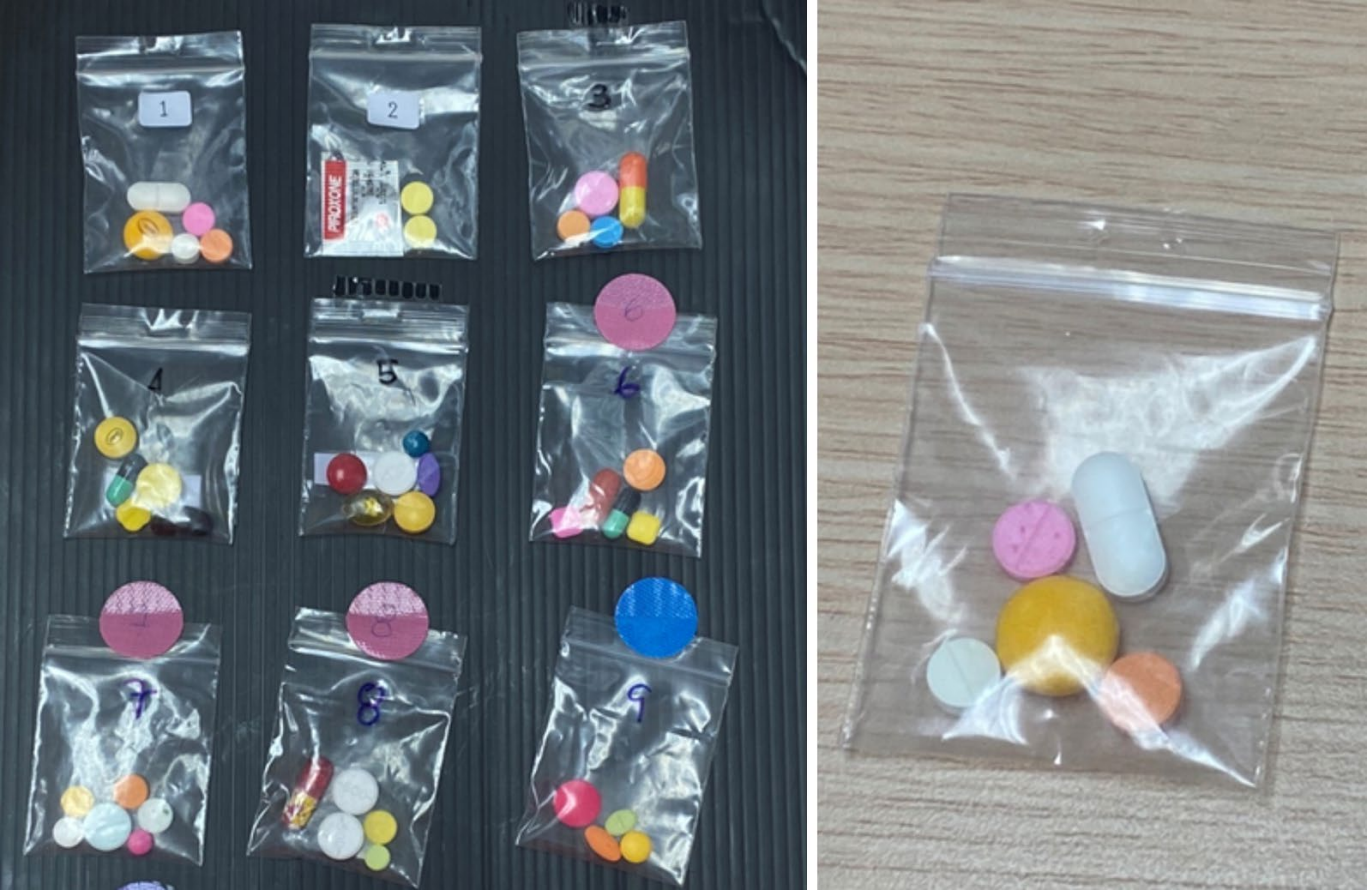
The representative images of steroids, painkillers, and NSAIDs are illegally available. Polypharmacy is common in the Northeast region of Thailand.

There was a positive association between increasing total WBC count and risk for CKD similar to what has been reported in US adults^31^. This finding hints at the possibility of systemic inflammation as the risk factor for CKD. It was found that polypharmacy was common in the study population. A group of steroids and painkillers such as NSAIDs, analgesic drugs, and muscle relaxants in a plastic pouch were easily available from peddlers or at groceries (Fig. 2). There was, however, no significant association between the use of over-the-counter drugs and NSAID and CKD in the study. This may be due to the avoidance of these drugs in the high-risk group and known CKD patients as advised by their primary care physicians or nephrologists.

The present study has some limitations. First, the frequency of an aged population was comparatively higher than in previous studies in Thailand and the study focused on rural areas of one province, therefore, the results may not be generalized nationwide. The population was to be analyzed as a study to confirm that they represented the entire northeast population of Thailand, however, selection bias occurred from the migration of villagers for jobs and study or refusal to participate in the study that could not be controlled. Secondly, a cross-sectional design of the study interpreted an association but not a causal effect between kidney damage markers and concomitant factors. The strength of this study was to demonstrate how a high CKD prevalence exists, the importance of screening, and the urgency to prevent and slow CKD progression in rural regions.

In conclusion, the results showed that CKD has developed as a significant public health problem in rural areas of Thailand such as that one out of every four has CKD. These important findings are consistent with previous Thai studies and higher due to an older age group of population and the use of different criteria for CKD screening. It is therefore recommended to exercise potential interventions to reduce the related risk factors established in this study.

## Methods

### Study design and subjects

This study was a part of a sub-project under Chronic Kidney Disease Prevention in the Northeast of Thailand (CKDNET) Project^32^ in which the population-based study was conducted in two rural sub-districts namely Don Chang and Khok Samran of Khon Kaen province, located in the Northeast Thailand from 2017 to 2019. The residents in 24 villages (of these sub-districts), willing to participate and meeting the inclusion criteria: aged ≥ 18 years and without psychopathic disorders were enrolled in the study. The two sub-districts were selected for the research because their population and socio-economic levels were representatives of northeastern Thailand. This was confirmed by analyzing the existing censuses of the Northeast Thailand, Khon Kaen province, Khok Samran, and Don Chang districts before starting the study (https://hdcservice.moph.go.th/hdc/reports/page.php?cat_id=ac4eed1bddb23d6130746d62d2538fd0)). The distributions of age and sex of population in these areas were quite similar with about 48% males and the highest proportion in age 40–49 years (Table S9). Also, the average personal monthly income in 2015 was below 10,000 Baht in both Northeast Thailand and Khon Kaen province (6790 vs. 7813 Baht) (https://www.nesdc.go.th/ewt_w3c/ewt_dl_link.php?nid=7787). Stratified cluster sampling was performed to enroll the participants as distributed in the censuses. In reality, however, the younger and early middle-aged villagers, especially the males, were out of their villages to study and/or work. Furthermore, they were less interested to participate in the study resulting in a selection bias with a higher proportion of females (65%) and the higher age group of 50–59 years as shown in Table S9.

### Sample size estimation

The sample size calculation was based on the objectives to evaluate prevalence and factors associated with CKD by stratified-cluster sampling and performing multiple logistic regression analyses. HT was used as the main risk factor of CKD and data from the Thai SEEK study were used as a reference in which the proportion of HT was 27.5% with the event rate of CKD in HT was 34.4% and in non-HT was 11.9%^10^. The multiple correlation coefficient between HT and the remaining covariates in multiple logistic regression was set as 0.8 and a design effect for stratified-cluster sampling was set as two times higher than simple random sampling. The sample size of 1700 subjects were required using the formula suggested by Hsieh, Block, and Larsen (1998) for a power of 90% and a targeted significance level of 0.05^33^. The dropout was set at 25%, therefore, the least total sample size was 2268 cases.

### Procedure and measurements

**Figure 3:**
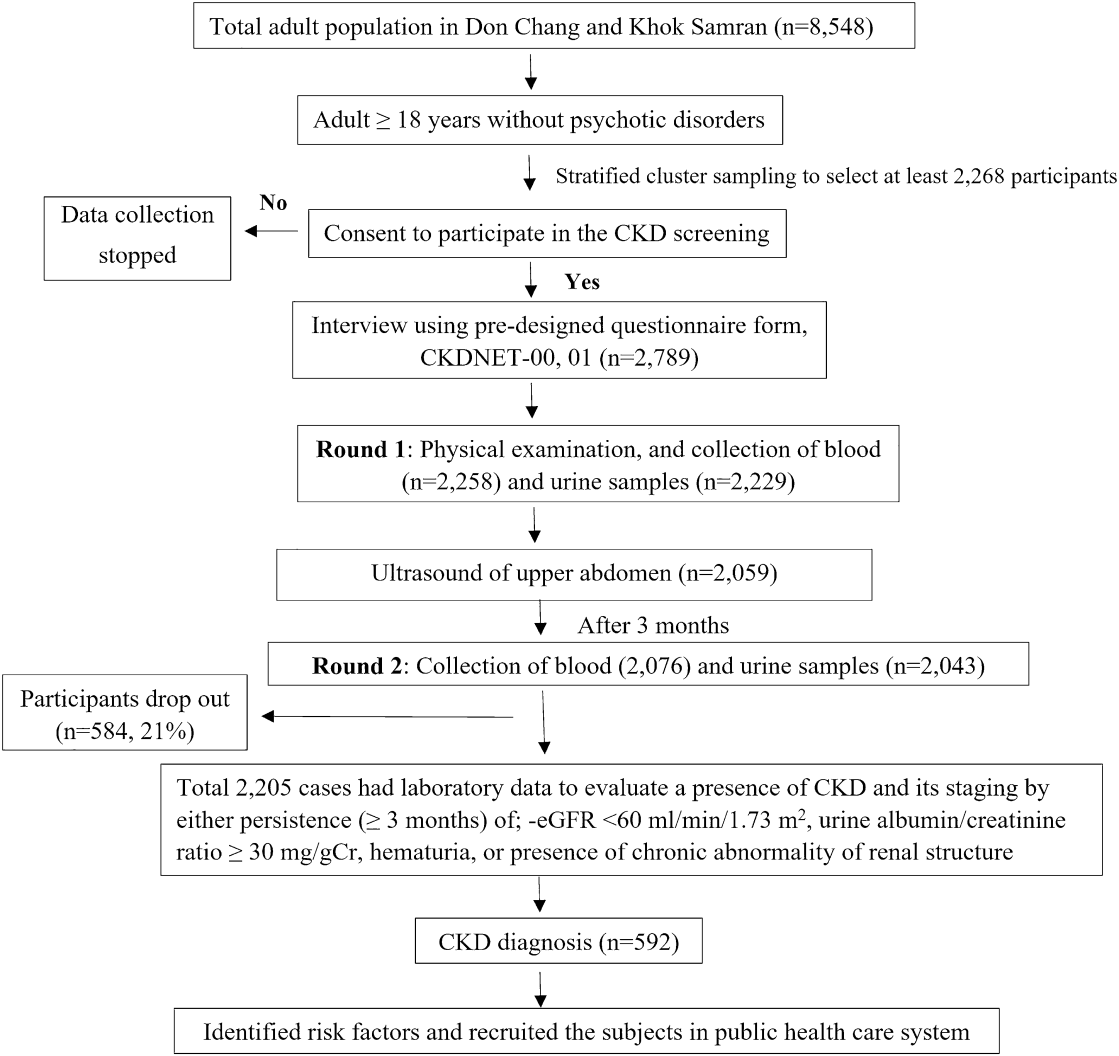
An outline of the CKD screening procedure. The participants were surveyed using a pre-designed questionnaire on their socio-economic status, lifestyle, and underlying diseases. Physical examinations and sample collections were performed twice following the KDIGO criteria. Upon being diagnosed with CKD, the participants were enrolled in the national public health care system.

An outline of the study procedures is illustrated in Fig. 3. All on-site screening was performed by the work-specific research team including trained post-graduate students, nurses, technicians, physicians, faculty members, and health volunteers. Data were obtained by interviewing the participants in their homes a month before the initial sample collection step with a pre-designed questionnaire on socio-demographic status that included age, sex, education level, monthly income, history of personal and family health, lifestyle (smoking, alcohol consumption, physical exercise, use of NSAIDs), dietary patterns and farming practices (use of insecticides, herbicides, etc.), underlying diseases and medications, and symptoms related to kidney diseases.

The first round of blood and urine sample collection was performed with prior notice to the participants to maintain 8–10 h of overnight fasting. First, arterial blood pressure was measured after resting for 5–10 min in a sitting position by a TERUMO digital blood pressure monitor ES-W100 (Terumo Corporation, Japan). The measurements were repeated in case of a hypertensive reading, and the average value of two measurements was used. Then, weight and height were measured and recorded in the examination form. Body composition analysis for fat, muscle, and water contents was also conducted using a bio-impedance machine (Fresenius Medical Care, Germany). Finally, blood was collected in vials with and without anticoagulants, as well as fresh morning spot urine was received in a sterile plastic container. Samples were then stored and transported at the controlled temperature of 4–10 °C to the central laboratory, Srinagarind Hospital, KKU within 6 h.

Only the participants who completed the socio-economic survey and got through the first round of blood and urine collections were continued to the next steps. Upper abdominal ultrasonography was performed after a month of sample collections, by three radiologists to investigate structural abnormalities in the kidneys. Any questionable findings were double-checked by another investigator. The participants with abnormalities in the first ultrasound but without other criteria of CKD were repeated after 12 weeks for confirmation. The inter-observer agreement (Cohen’s kappa) among these three operators performing in 102 participants was 0.83 (small size of kidneys), 0.88 (renal stones), 0.79 (hydronephrosis), and 0.78 (parenchymatous changes).

Blood and urine specimen collections were repeated after 3 months for the round 2. Urinary albumin to creatinine ratio (UACR, mg/g) and eGFR was calculated using the CKD-EPI (Chronic Kidney Disease Epidemiology Collaboration) Eq. ^34^. Also, the presence of red blood cells in urine was investigated under a microscope. The participants were then categorized as CKD if matched with either of the following criteria for 3 months or longer: 1) eGFR < 60 ml/min/​​1.73 m^2^, or 2) presence of any kidney damage; UACR ≥ 30 mg/g in two consecutive samples; ultrasonography examination indicating at least one of the following chronic kidney conditions and structural abnormalities: parenchymatous change of kidney, renal stones, complex cysts and polycystic disease (excluding simple cysts), small kidney size (< 8.5 cm.) with parenchymal change, presence of hydronephrosis, tumor in the kidneys, unilateral kidney (post nephrectomy, renal agenesis or renal atrophy), and presence of urine red blood cells (RBC) ≥ 3–5 cells/high power field (HPF) in two consecutive samples.

### Variability of interest and definitions

CKD was defined based on the KDIGO criteria as the presence of either one or more parameters of renal damage or reduced estimated glomerular filtration rate (eGFR) less than 60 ml/min/1.73 m^2^ for 3 months or longer^35^. The classification of CKD was based on eGFR levels as stage 1: ≥ 90, stage 2: 60–89, stage 3a: 45–59, stage 3b: 30–44, stage 4: 15–29, and stage 5: < 15 ml/min/1.73 m^2^. UACR levels were divided into 3 categories as follows: A1 < 30, A2 30–300 and A3 > 300 mg/g, when UACR ≥ 30 mg/g was considered as albuminuria. Hyperuricemia was defined as serum acid uric > 7 mg/dL for males and > 6 mg/dL for females. DM was defined by diagnosed DM from physicians and being on medication, or levels of fasting plasma glucose (FPG) ≥ 126 mg/dL or hemoglobin A1c ≥ 6.5% for two times. HT was considered when subjects had a persistent systolic blood pressure (SBP) ≥ 140 mm Hg, and diastolic blood pressure (DBP) ≥ 90 mm Hg or were diagnosed as hypertensive by physicians and being on medication. Anemia was diagnosed if the participants had hemoglobin levels < 13 g/dL in males and < 12 g/dL in females and hypercholesterolemia was defined as low-density lipoprotein (LDL)-cholesterol ≥ 130 mg/dL or on cholesterol-lowering medicines. The participants were considered to have awareness of HT, DM, CKD, and other related conditions/diseases if they had an earlier diagnosis by physicians.

Participants’ education was categorized into 7 levels: no schooling, primary school, middle school, high school/vocational certificate, diploma/high or vocational school, bachelor’s degree, and postgraduate. Economic conditions were grouped into 2 levels based on monthly income: ≤ 10,000 Baht and ≥ 10,000 Baht. Physical exercise was measured in terms of frequency/week and exercise duration. The body mass index (BMI) was calculated as weight (kg)/ height (m^2^). Then, the BMI classifications for Asian adults from the Regional Office for the Western Pacific (WPRO) of the World Health Organization (WHO) were used, and subsequently, the subjects were categorized into underweight (< 18.5), normal (18.5–22.9), overweight (23–24.9), class I obesity (25–29.9), and class II obesity (≥ 30)^36^.

### Laboratory assessment

Blood was analyzed for red blood cell count, hematocrit, platelets (DC sheath flow method), white blood cells (WBC), WBC differentiation (fluorescent flow cytometry), and hemoglobin (cyanide-free SLS method) using an XS-800i hematology analyzer (Sysmex Corporation, Japan) while serums were investigated for alanine transferase (IFCC method without pyridoxal phosphate), uric acid (enzymatic colorimetric method), fasting blood sugar (kinetic reaction: HK/G6PDH), creatinine (enzymatic method standardized against Isotope Dilution Mass Spectrometry, IDMS) and LDL-Cholesterol (direct assay) by Cobas 6000 analyzer (Roche Diagnostics, USA). Urine was assessed for creatinine (enzymatic method) and microalbumin (immunoturbidimetric method) with the Cobas 8000 analyzer series (502 and 702 modules, Roche Diagnostics, USA) as well as routine urinalysis performed using flow cell digital imaging in the iQ200 urine microscopy analyzer (Beckman Coulter, Inc., USA).

### Ethics consideration

The study protocol conformed to the ethical guidelines of the 1975 Declaration of Helsinki that was approved by the Ethics Committee for Human Research, Faculty of Medicine, Khon Kaen University (KKU), Thailand (HE 601,035). All the participants provided written informed consent before the study.

### Statistical analyses

Statistical analyses were performed using STATA version 17.0 and a *P*-value < 0.05 was considered statistical significance. Data were presented as mean ± SD, median (interquartile range; IQR), or percentage. Inter-group comparisons were performed by the Chi-square test or Fisher’s Exact test for categorical variables and Student’s t-test, Mann–Whitney U-test for continuous variables, as appropriate. A logistic regression analysis was applied to predict an association between CKD and relevant covariates, and Odds Ratios (ORs) were reported with 95% confidential intervals (CIs). The ORs that accounted for the effects in univariable analysis with a *P*-value < 0.05 were entered into a multivariate logistic regression model and the interaction effects were evaluated. In the current study, the magnitude and severities of anemia were higher than expectation from CKD complications and some dependent variables were affected by anemia that needed adjustment for the demonstration of the real effect on those variables. Therefore, anemia as one of the independent variables was added. Data, however, were also analyzed by excluding anemia from multivariable analysis. Additionally, two models were investigated; Model 1 is better in terms of biostatistics analysis because data of the dependent variables were input as continuous data therefore, resulting in a greater amount and diversity of data compared to the Model 2, in which data were input as categorical data, i.e., DM, HT, Anemia, Hyperuricemia, leucocytosis, and hyperlipidemia. Some studies, however, focused on these categorical variables as the risk factors rather than detailing with them as individual data, therefore 2 models for benefit in comparison with the other studies were performed.

A priori target was set for an acceptable level of missing data as < 5% and used the multiple imputation method for handling the missing data. These missing data were replaced with a set of predicted values imputed from the other variables of existing data which contained the natural variability and uncertainty of the right values; then multiple imputed data sets were combined and repeatedly analyzed to produce the final single overall analysis result.

## Supplementary Information

Supplementary Information 1.

Supplementary Information 2.

## Data Availability

The datasets generated in the current study are available from the corresponding author on reasonable request.
