# Supplementary material for: High prevalence of chronic kidney disease and its related risk factors in rural areas of Northeast Thailand
Source: Sci Rep. 2022 Oct 28;12:18188. doi: 10.1038/s41598-022-22538-w (PMC9616930; doi:10.1038/s41598-022-22538-w)
Supplement: Supplementary file 1 — Supplementary Information 1. [file 41598_2022_22538_MOESM1_ESM.docx]

**Supplementary Materials**

**High prevalence of chronic kidney disease and its related risk factors in rural areas of Northeast Thailand**

Ubon Cha’on ^1,2^, Patcharaporn Tippayawat ^2,3^, Nattaya Sae-ung ^2,3^, Porntip Pinlaor ^2,3^, Wichien Sirithanaphol^4^, Ampornpan Theeranut^5^, Kriang Tungsanga^6,7^, Prathana Chowchuen^8^, Amod Sharma^2^, Supakit Boonlakron^9^, Sirirat Anutrakulchai* ^2,10^

^1^Department of Biochemistry, Faculty of Medicine, Khon Kaen University, Thailand

^2^Chronic Kidney Disease Prevention in the Northeast of Thailand (CKDNET) Project, Khon

Kaen University, Thailand

^3^Faculty of Associated Medical Sciences, Khon Kaen University, Thailand

^4^Department of Surgery, Faculty of Medicine, Khon Kaen University, Thailand

^5^Faculty of Nursing, Khon Kaen University, Thailand

^6^Department of Medicine, Faculty of Medicine, King Chulalongkorn Memorial Hospital,

Chulalongkorn University, Bangkok, Thailand.

^7^Bhumirajanagarindra Kidney Institute, Bangkok, Thailand.

^8^Department of Radiology, Faculty of Medicine, Khon Kaen University, Thailand

^9^Department of Medical Services, Institute of Dermatology, Ministry of Public Health,

Bangkok, Thailand

^10^Department of Medicine, Faculty of Medicine, Khon Kaen University, Thailand

*Corresponding author:

Sirirat Anutrakulchai

Division of Nephrology, Department of Internal Medicine,

Faculty of Medicine, Khon Kaen University, Khon Kaen, 40002, Thailand

Email: [sirirt_a@kku.ac.th](mailto:sirirt_a@kku.ac.th)

**Supplementary Tables: 9**

**Supplementary Figures: 4**

**Supplementary tables**

**Table S1: A comparison between the total participants, those who completed the study and dropped out from the study**

| **Demographic data** | **Total participants enrolled in the study (n=2,789)** | **Total participants who completed the study (n=2,205)** | **Participants who dropped out from the study (n=584)** | **P-value*** |
| --- | --- | --- | --- | --- |
| Age, years (mean ± SD)  Age distribution, n (%)  18-29  30-39  40-49  50-59  60-69  ≥ 70 | 56.9 ± 12.8  101 (3.62)  129 (4.63)  476 (17.1)  895 (32.1)  741 (26.6)  447 (16.0) | 57.8 ±11.7  41 (1.86)  83 (3.76)  359 (16.3)  742 (33.7)  629 (28.5)  351 (15.9) | 53.2 ± 16.0  60 (10.3)  46 (7.88)  117 (20.0)  153 (26.2)  112 (19.2)  96 (16.4) | <0.001  < 0.001 |
| Sex: Male (n, %) | 981 (35.2) | 735 (33.3) | 246 (42.1) | < 0.001 |
| Education, n (%) |  |  |  | < 0.001 |
| Did not study | 18 (0.65) | 12 (0.54) | 6 (1.03) |  |
| Primary school | 2,139 (76.7) | 1,741 (79.0) | 398 (68.2) |  |
| Middle school | 239 (8.57) | 175 (7.94) | 64 (11.0) |  |
| High school/Vocational | 258 (9.25) | 192 (8.71) | 66 (11.3) |  |
| Diploma/High Vocational | 60 (2.15) | 36 (1.63) | 24 (4.11) |  |
| Bachelor’s degree | 66 (2.37) | 44 (2.00) | 22 (3.77) |  |
| Postgraduate | 9 (0.32) | 5 (0.23) | 4 (0.68) |  |
| Occupation |  |  |  |  |
| Farmer | 1,651 (59.2) | 1,401 (63.5) | 250 (42.8) | < 0.001 |
| Trader | 132 (4.73) | 89 (4.04) | 43 (7.36) |  |
| Jobs in factories, companies | 75 (2.69) | 41 (1.86) | 34 (5.82) |  |
| Government/Enterprise | 40 (1.43) | 33 (1.50) | 7 (1.20) |  |
| Student | 24 (0.86) | 8 (0.36) | 16 (2.74) |  |
| Other | 357 (12.8) | 248 (11.3) | 109 (18.7) |  |
| Unemployed | 510 (18.3) | 385 (17.5) | 125 (21.4) |  |
| Monthly income (Baht), n (%) |  |  |  | < 0.001 |
| <10,000  >10,000 | 2,476 (88.8)  313 (11.2) | 1,998 (90.6)  207 (9.39) | 478 (81.8)  106 (18.2) |  |

* P-values compared between the total participants who completed the study and participants who dropped out from the study

**Table S2: Distribution of CKD cases in each age group and sex**

| **Age groups (years)** | **Both sexes** | | | **Male** | | | **Female** | | |
| --- | --- | --- | --- | --- | --- | --- | --- | --- | --- |
|  | **Total cases**  **n= 2,205**  **(100%)** | **CKD cases (n= 592)** | | **Total cases**  **n=735**  **(100%)** | **CKD cases (n=234)** | | **Total cases**  **n=1,470**  **(100%)** | **CKD cases (n=358)** | |
|  |  | **% of total CKD cases** | **% of each**  **age group** |  | **% of total CKD cases** | **% of each**  **age group** |  | **% of total CKD cases** | **% of each**  **age group** |
| 18-29 | 41 (1.86) | 0 (0.00) | 0.00 | 14 (1.90) | 0 (0.00) | 0.00 | 27 (1.84) | 0 (0.00) | 0.00 |
| 30-39 | 83 (3.76) | 12 (2.03) | 14.46 | 20 (2.72) | 4 (1.71) | 20.00 | 63 (4.29) | 8 (2.23) | 12.70 |
| 40-49 | 359 (16.28) | 45 (7.60) | 12.53 | 89 (12.11) | 15 (6.41) | 16.85 | 270 (18.37) | 30 (8.38) | 11.11 |
| 50-59 | 742 (33.65) | 143 (24.16) | 19.27 | 244 (33.20) | 58 (24.79) | 23.77 | 498 (33.88) | 85 (23.74) | 17.07 |
| 60-69 | 629 (28.53) | 207 (34.97) | 32.91 | 236 (32.11) | 87 (37.18) | 36.86 | 393 (26.73) | 120 (33.52) | 30.53 |
| ≥ 70 | 351 (15.92) | 185 (31.25) | 52.71 | 132 (17.96) | 70 (29.91) | 53.03 | 219 (14.90) | 115 (32.12) | 52.51 |

CKD, Chronic Kidney Disease

**Table S3: Comparison of results for various CKD criteria among round 1, round 2 and both 2 rounds in the study population**

|  | **Round 1** | | **Round 2** | | **CKD cases after 2 rounds** | |
| --- | --- | --- | --- | --- | --- | --- |
|  | **Total cases**  **investigated**  **(n, %)** | **Number of abnormal tests**  **(n, %)** | **Total cases**  **investigated (n, %)** | **Number of abnormal tests (n, %)** | **Total cases**  **investigated**  **(n, %)** | **Number of**  **abnormal tests***  **(n, %)** |
|  |  |  |  |  |  |  |
| **Single criterion** |  |  |  |  |  |  |
| eGFR < 60 ml/mim/1.73 m^2^ | 2,258 (100) | 267 (11.82) | 2,076 (100) | 251 (12.09) | 2,076 (100) | 212 (10.21) |
| Microalbuminuria (≥ 30 mg/g Cr) | 2,229 (100) | 384 (17.23) | 2,043 (100) | 367 (17.96) | 2,022 (100) | 253 (12.51) |
| Hematuria (RBC > 3 cells/HPF) | 2,229 (100) | 196 (8.79) | 2,042 (100) | 125 (6.12) | 2,019 (100) | 60 (2.97) |
| Structural abnormalities detected  by ultrasound** | 2,059 (100) | 333 (16.17) | NA | NA | 2,059 (100) | 333 (16.17) |
| **Multiple criteria** |  |  |  |  |  |  |
| eGFR and/or microalbuminuria | 2,258 (100) | 529 (23.43) | 2,078 (100) | 512 (24.64) | 2,144 (100)^#^ | 384 (17.91) |
| eGFR and/or microalbuminuria  and/or hematuria | 2,258 (100) | 640 (28.34) | 2,078 (100) | 575 (27.67) | 2,144 (100) ^#^ | 413 (19.26) |
| eGFR and/or microalbuminuria  and/or hematuria and/or  abnormal ultrasound** | 2,258 (100) | 788 (34.90) | NA | NA | 2,205 (100) ^##^ | 592 (26.85) |

* The participants had complete laboratory data to evaluate the presence of CKD and its staging by at least one criterion of persistence (≥ 3 months) of; -eGFR <60 ml/min/1.73 m^2^, urine albumin/creatine ratio (ACR) ≥ 30 mg/gCr, hematuria, or presence of chronic abnormality of renal structure.

** The participants had ultrasound 1 time except for the 102 cases in total 179 cases without other criteria of CKD were repeated to confirm the abnormalities

^#^ Including; (1) the participants who have assessed laboratory data for two rounds (n=2071, which excluded 7 patients in round 2 because of undetermined CKD status, i.e., 2 times of their eGFR levels were > 60 ml/min/1.73 m^2^, but either ACR or urine RBC was measured one time and the levels were abnormal), (2) one round with all eGFR, albuminuria, and urine RBC levels were not compatible with the criteria of CKD (defined as non-CKD, n=73)

^##^ Same including as^#^ and add 61 subjects performed ultrasound with one round of laboratories (10 cases defined as CKD because of abnormal ultrasound and 51 cases defined as non-CKD because of normal ultrasound and no compatible with CKD criteria of one round laboratory)

**Table S4: Details of 333 CKD cases who had abnormal renal ultrasound as the criterion of CKD**

| **CKD cases who had abnormal ultrasound** | **Renal parenchymatous change, n (%)** | | **Small size of kidneys** | | **Renal stone** | **Hydronephrosis** | **Polycystic kidneys** | **Complex**  **cysts** | **Renal AML** |
| --- | --- | --- | --- | --- | --- | --- | --- | --- | --- |
|  | **One kidney** | **Bilateral kidneys** | **One kidney** | **Bilateral kidneys** |  |  |  |  |  |
| With 2 rounds of other CKD criteria (n=154, 100%) | 38  (24.68) | 65  (42.21) | 32  (20.78) | 11  (7.14) | 52  (33.77) | 21  (13.64) | 2  (1.30) | 4  (2.60) | 0  (0.00) |
| With 1 round of other CKD criteria (n=56, 100%) | 15  (26.79) | 23  (41.07) | 8  (14.29) | 3  (5.36) | 25  (44.64) | 4  (7.14) | 2  (3.57) | 2  (3.57) | 0  (0.00) |
| No other CKD criteria (n=123, 100%) | 36  (29.27) | 43  (34.96) | 16  (13.01) | 6  (4.88) | 46  (37.40) | 15  (12.20) | 1  (0.81) | 4  (3.25) | 3  (2.44) |
| Total (n=333, 100%) | 89 (26.73) | 131 (39.34) | 56 (16.82) | 20 (6.01) | 123 (36.94) | 40 (12.01) | 5 (1.50) | 10 (3.00) | 3 (0.90) |

CKD, Chronic Kidney Disease; AML, Angiomyolipoma

**Table S5: Prevalence of CKD and CKD staging by using different GFR estimating equations (total cases, n=2205)**

| **GFR estimating equations** | **CKD prevalence n (%)** | **CKD staging** | | | | | |
| --- | --- | --- | --- | --- | --- | --- | --- |
|  |  | **1** | **2** | **3a** | **3b** | **4** | **5** |
| CKD-EPI* | 592 (26.8) | 162 (7.35) | 198 (9.00) | 133 (6.03) | 62 (2.81) | 31 (1.41) | 6 (0.27) |
| MDRD** | 614 (27.8) | 122 (5.53) | 224 (10.2) | 139 (6.30) | 93 (4.22) | 30 (1.36) | 6 (0.27) |
| Thai-GFR 1*** | 567 (25.7) | 189 (8.57) | 203 (9.21) | 105 (4.76) | 42 (1.90) | 22 (1.00) | 6 (0.27) |
| Thai-GFR 2**** | 588 (26.7) | 125 (5.67) | 259 (11.8) | 136 (6.17) | 55 (2.49) | 11(0.50) | 2 (0.09) |

CKD, Chronic Kidney Disease; CKD-EPI, Chronic Kidney Disease Epidemiology Collaboration; MDRD, Modification of Diet in Renal Disease

GFR, Glomerular Filtration Rate

*Female: Scr ≤ 0.7, eGFR = 144 x (Scr/0.7) ^-0.329^ x (0.993)^Age^

> 0.7, eGFR = 144 x (Scr/0.7) ^-1.209^ x (0.993)^Age^

Male: Scr ≤ 0.9, eGFR = 141 x (Scr/0.9) ^-0.411^ x (0.993)^Age^

> 0.9, eGFR = 141 x (Scr/0.9)^-1.209^ x (0.993)^Age^

**175 x (Scr) ^-1.154^ x (Age)^-0.203^ x (0.742 if female) x (1.212 if black)

***175 × Cr (Enz) ^(-1.154)^ × Age ^(-0.203)^ × 0.742 (if female) × 1.129

****375.5 × Cr (Enz) ^(-0.848)^ × Age^(-0.364)^ × 0.712 (if female)

**Table S6: The analysis of CKD risk factors excluding anemia and hemoglobin variables in the models**

| Factors | Crude OR  (95% CI) | P-value | Model 1 | | Model 2 | |
| --- | --- | --- | --- | --- | --- | --- |
|  |  |  | Adjusted OR  (95 % CI) | p-value | Adjusted OR  (95% CI) | p-value |
| Age (every 1-year increase)  Male  Low monthly income (<10,000 Baht)  Less education (none plus primary school)  Unemployed status  Smoking habits  No  Quit  Till now  NSAID use  Never used  Used to take  Still taking  Alcohol consumption  Do not drink  Used to drink  Currently drinking | 1.07 (1.06-1.09)  1.45 (1.19-1.76)  1.83 (1.26-2.66)  2.19 (1.67-2.86)  2.51 (2.00-3.16)  1  1.39 (1.04-1.86)  1.40 (1.06-1.85)  1  0.80 (0.63-1.02)  0.72 (0.55-0.93)  1  1.31 (0.98-1.76)  0.79 (0.63-0.99) | <0.001  <0.001  0.001  <0.001  <0.001  0.011  0.024  0.018  0.019  0.074  0.013  0.010  0.069  0.044 | 1.05 (1.04-1.07)  0.96 (0.67-1.38)  0.89 (0.58-1.37)  0.93 (0.66-1.29)  1.42 (1.07-1.89)  1  0.85 (0.54-1.33)  1.43 (0.93-2.19)  1  1.02 (0.77-1.35)  0.81 (0.60-1.09)  1  0.87 (0.58-1.32)  0.74 (0.54-1.01) | <0.001  0.813  0.601  0.655  0.017  0.468  0.106  0.897  0.168  0.524  0.059 | 1.05 (1.04-1.06)  1.69 (1.18-2.41)  0.89 (0.58-1.37)  0.97 (0.70-1.36)  1.36 (1.02-1.81)  1  0.80 (0.51-1.26)  1.43 (0.93-2.19)  1  0.92 (0.69-1.22)  0.79 (0.59-1.06)  1  0.82 (0.54-1.24)  0.76 (0.55-1.04) | <0.001  0.004  0.592  0.871  0.037  0.333  0.102  0.546  0.118  0.347  0.091 |
| Drinking water (every increase of 500 ml a day) | 0.93 (0.86-0.98) | 0.009 | 0.98 (0.92-1.04) | 0.496 | 0.98 (0.92-1.04) | 0.448 |
| Underweight (BMI <18.5 kg/m^2^)  BMI (every 1 kg/m^2^ increase) | 1.85 (1.24-2.76)  0.98 (0.96-1.005) | 0.003  0.123 | 0.98 (0.95-1.006) | 0.128 | 1.72 (1.08-2.73) | 0.022 |
| DM  Fasting plasma glucose (every 1-mg/dL increase) | 3.04 (2.45-3.77)  1.007 (1.006-1.009) | <0.001  <0.001 | 1.007 (1.005-1.009) | <0.001 | 2.21 (1.70-2.87) | <0.001 |
| HT without DM  HT with DM  SBP (every 1-mmHg increase) | 2.49 (1.96-3.16)  5.39 (4.14-7.04)  1.024 (1.019-1.030) | <0.001  <0.001  <0.001 | 1.010 (1.003-1.016) | 0.003 | 1.81 (1.42-2.30) | <0.001 |
| Hyperuricemia  Serum uric acid (every 1-mg/dL increase) | 3.71 (2.99-4.60)  1.52 (1.42-1.63) | <0.001  <0.001 | 1.44 (1.32-1.56) | <0.001 | 2.74 (2.14-3.51) | <0.001 |
| Leukocytosis  WBC count (every 1,000-cells/mL increase)  Hyperlipidemia  LDL-cholesterol (every 1-mg/dL increase) | 1.66 (1.26-2.20)  1.16 (1.11-1.22)  0.66 (0.55-0.80)  0.994 (0.992-0.997) | <0.001  <0.001  <0.001  <0.001 | 1.13 (1.07-1.20)  0.996 (0.993-0.999) | <0.001  0.007 | 1.40 (1.0002-1.95)  0.75 (0.60-0.94) | 0.050  0.012 |

**Table S7: Factors associated with CKD stage 3-5 (including anemia and hemoglobin variables in the models)**

| Factors | Crude OR  (95% CI) | p-value | Model 1 | | Model 2 | |
| --- | --- | --- | --- | --- | --- | --- |
|  |  |  | Adjusted OR  (95 % CI) | p-value | Adjusted OR  (95% CI) | p-value |
| Age (every 1-year increase)  Male  Low monthly income (<10,000 Baht)  Less education (none plus primary school)  Unemployed status  Smoking habits  No  Quit  Till now  NSAID use  Never used  Used to take  Still taking  Alcohol consumption  Do not drink  Used to drink  Continuously drinking | 1.16 (1.14-1.18)  1.28 (0.97-1.69)  4.27 (1.87-9.74)  4.45 (2.56-7.71)  4.29 (3.22-5.73)  1  1.40 (0.94-2.08)  0.90 (0.58-1.40)  1  0.71 (0.50-1.03)  0.64 (0.43-0.95)  1  1.36 (0.92-2.00)  0.38 (0.25-0.58) | <0.001  0.086  0.001  <0.001  <0.001  0.212  0.096  0.645  0.027  0.068  0.027  <0.001  0.121  <0.001 | 1.14 (1.11-1.17)  1.42 (0.76-2.66)  0.68 (0.24-1.95)  0.79 (0.38-1.65)  1.23 (0.78-1.93)  1  0.62 (0.28-1.39)  1.40 (0.63-3.09)  1  0.97 (0.57-1.65)  0.95 (0.54-1.67)  1  0.93 (0.45-1.91)  0.44 (0.22-0.85) | <0.001  0.276  0.469  0.532  0.380  0.246  0.409  0.911  0.860  0.848  0.015 | 1.14 (1.11-1.17)  2.46 (1.36-4.46)  0.80 (0.28-2.27)  0.81 (0.40-1.68)  1.12 (0.72-1.74)  1  0.55 (0.26-1.18)  1.27 (0.59-2.72)  1  0.86 (0.52-1.44)  0.89 (0.51-1.53)  1  0.90 (0.45-1.78)  0.51 (0.27-0.96) | <0.001  0.003  0.674  0.577  0.622  0.123  0.544  0.573  0.669  0.763  0.038 |
| Drinking water (every increase of 500 ml a day) | 0.81 (0.75-0.89) | <0.001 | 0.99 (0.89-1.11) | 0.882 | 0.99 (0.89-1.09) | 0.793 |
| Underweight (BMI <18.5 kg/m^2^)  BMI (every 1 kg/m^2^ increase) | 2.37 (1.45-3.87)  0.96 (0.93-0.99) | 0.001  0.012 | 1.005 (0.95-1.06) | 0.861 | 1.78 (0.88-3.61) | 0.109 |
| DM  Fasting plasma glucose (every 1-mg/dL increase) | 3.44 (2.59-4.57)  1.005 (1.003-1.007) | <0.001  <0.001 | 1.008 (1.005-1.012) | <0.001 | 2.81 (1.81-4.37) | <0.001 |
| HT without DM  HT with DM  SBP (every 1-mmHg increase) | 4.01 (2.84-5.65)  7.69 (5.46-10.8)  1.03 (1.020-1.04) | <0.001  <0.001  <0.001 | 1.005 (0.99-1.016) | 0.346 | 2.16 (1.42-3.27) | <0.001 |
| Anemia  Hemoglobin (every 1-g/dL increase) | 7.57 (5.56-10.3)  0.53 (0.48-0.59) | <0.001  <0.001 | 0.58 (0.50-0.67) | <0.001 | 4.50 (3.02-6.70) | <0.001 |
| Hyperuricemia  Serum uric acid (every 1-mg/dL increase) | 11.0 (8.16-14.9)  2.30 (2.08-2.56) | <0.001  <0.001 | 2.33 (2.02-2.69) | <0.001 | 9.42 (6.36-14.0) | <0.001 |
| Leukocytosis  WBC count (every 1,000-cells/mL increase)  Hyperlipidemia  LDL-cholesterol (every 1-mg/dL increase) | 1.71 (1.18-2.49)  1.14 (1.07-1.22)  0.50 (0.38-0.67)  0.990 (0.986-0.994) | 0.005  <0.001  <0.001  <0.001 | 1.14 (1.04-1.25)  0.995 (0.990-1.000) | 0.005  0.054 | 1.50 (0.86-2.60)  0.75 (0.50-1.13) | 0.149  0.166 |

**Table S8: Factors associated with CKD stage 3-5 (excluding anemia and hemoglobin variables in the models)**

| Factors | Crude OR  (95% CI) | p-value | Model 1 | | Model 2 | |
| --- | --- | --- | --- | --- | --- | --- |
|  |  |  | Adjusted OR  (95% CI) | p-value | Adjusted OR  (95% CI) | p-value |
| Age (every 1-year increase)  Male  Low monthly income (<10,000 Baht)  Less education (none plus primary school)  Unemployed status  Smoking habits  No  Quit  Till now  NSAID use  Never used  Used to take  Still taking  Alcohol consumption  Do not drink  Used to drink  Currently drinking | 1.16 (1.14-1.18)  1.28 (0.97-1.69)  4.27 (1.87-9.74)  4.45 (2.56-7.71)  4.29 (3.22-5.73)  1  1.40 (0.94-2.08)  0.90 (0.58-1.40)  1  0.71 (0.50-1.03)  0.64 (0.43-0.95)  1  1.36 (0.92-2.00)  0.38 (0.25-0.58) | <0.001  0.086  0.001  <0.001  <0.001  0.212  0.096  0.645  0.027  0.068  0.027  <0.001  0.121  <0.001 | 1.15 (1.12-1.18)  0.77 (0.43-1.38)  0.65 (0.24-1.76)  0.79 (0.39-1.60)  1.32 (0.86-2.04)  1  0.65 (0.30-1.38)  1.08 (0.50-2.32)  1  1.17 (0.71-1.95)  1.00 (0.59-1.70)  1  0.91 (0.46-1.80)  0.37 (0.19-0.70) | <0.001  0.387  0.399  0.510  0.207  0.260  0.840  0.538  0.998  0.780  0.002 | 1.15 (1.12-1.18)  2.31 (1.30-4.11)  0.87 (0.32-2.33)  0.88 (0.43-1.77)  1.13 (0.74-1.73)  1  0.59 (0.28-1.23)  1.24 (0.59-2.60)  1  0.98 (0.60-1.61)  0.81 (0.48-1.35)  1  0.86 (0.45-1.66)  0.44 (0.24-0.82) | <0.001  0.004  0.778  0.711  0.561  0.160  0.570  0.945  0.413  0.649  0.009 |
| Drinking water (every increase of 500 ml a day) | 0.81 (0.75-0.89) | <0.001 | 0.97 (0.87-1.08) | 0.602 | 0.98 (0.89-1.09) | 0.761 |
| Underweight (BMI <18.5 kg/m^2^)  BMI (every 1 kg/m^2^ increase) | 2.37 (1.45-3.87)  0.96 (0.93-0.99) | 0.001  0.012 | 0.97 (0.92-1.01) | 0.164 | 2.02 (1.01-4.03) | 0.046 |
| DM  Fasting plasma glucose (every 1-mg/dL increase) | 3.44 (2.59-4.57)  1.005 (1.003-1.007) | <0.001  <0.001 | 1.008 (1.005-1.011) | <0.001 | 3.12 (2.04-4.76) | <0.001 |
| HT without DM  HT with DM  SBP (every 1-mmHg increase) | 4.01 (2.84-5.65)  7.69 (5.46-10.8)  1.03 (1.020-1.04) | <0.001  <0.001  <0.001 | 1.006 (0.996-1.017) | 0.229 | 2.14 (1.44-3.20) | <0.001 |
| Hyperuricemia  Serum uric acid (every 1-mg/dL increase) | 11.0 (8.16-14.9)  2.30 (2.08-2.56) | <0.001  <0.001 | 2.41 (2.09-2.77) | <0.001 | 9.74 (6.66-14.2) | <0.001 |
| Leukocytosis  WBC count (every 1,000-cells/mL increase)  Hyperlipidemia  LDL-cholesterol (every 1-mg/dL increase) | 1.71 (1.18-2.49)  1.14 (1.07-1.22)  0.50 (0.38-0.67)  0.990 (0.986-0.994) | 0.005  <0.001  <0.001  <0.001 | 1.13 (1.03-1.24)  0.991 (0.985-0.996) | 0.008  <0.001 | 1.47 (0.87-2.46)  0.61 (0.41-0.89) | 0.149  0.011 |

**Table S9: The details of the census during 2016-2019 in Northeast Thailand, Khon Kaen, and the study setting areas**

| Years | Demography | Northeast Thailand | Khon Kaen | Khok Samran and Don Chang | This study period (2017-2019)** |
| --- | --- | --- | --- | --- | --- |
| 2016 | -Total adult population*, n (%)  Male  Female  -Age distribution (years), n (%)  20-29  30-39  40-49  50-59  60-69  ≥ 70 | 11,778,158 (100.0)  5,658,967 (48.0)  6,119,191 (52.0)  2,058,610 (17.5)  2,085,112 (17.7)  2,660,894 (22.6)  2,321,989 (19.7)  1,554,663 (13.2)  1,096,890 (9.31) | 1,017,011 (100.0)  482,803 (47.5)  534,208 (52.5)  191,957 (18.9)  174,356 (17.1)  224,014 (22.0)  203,325 (20.0)  136,901 (13.5)  86,458 (8.50) | 8,548 (100.0)  4,115 (48.1)  4,433 (51.9)  1,516 (17.8)  1,471 (17.2)  1,931 (22.6)  1,802 (21.1)  1,140 (13.3)  688 (8.04) | -Total participants enrolled in the study (n =2,789 cases, 100 %)  Male 981 (35.2)  Female 1,808 (64.8)  -Age distribution, n (%)  18-29 101 (3.62)  30-39 129 (4.63)  40-49 476 (17.1)  50-59 895 (32.1)  60-69 741 (26.6)  ≥ 70 447 (16.0)  -Total participants who completed the study (n = 2,205 cases, 100%)  Male 735 (33.3)  Female 1,470 (66.7)  -Age distribution, n (%)  18-29 41 (1.86)  30-39 83 (3.76)  40-49 359 (16.3)  50-59 742 (33.7)  60-69 629 (28.5)  ≥ 70 351 (15.9) |
| 2017 | -Total adult population*, n (%)  Male  Female  -Age distribution (years), n (%)  20-29  30-39  40-49  50-59  60-69  ≥ 70 | 11,926,958 (100.0)  5,736,573 (48.1)  6,190,385 (51.9)  2,130,717 (17.9)  2,011,243 (16.9)  2,621,744 (22.0)  2,371,820 (19.9)  1,619,135 (13.6)  1,172,299 (9.83) | 1,028,448 (100.0)  488,611 (47.5)  539,837 (52.5)  198,092 (19.3)  168,793 (16.4)  218,011 (21.2)  205,743 (20.0)  142,219 (13.8)  95,590 (9.29) | 8,737 (100.0)  4,199 (48.1)  4,538 (51.9)  1,564 (17.9)  1,441 (16.5)  1,918 (22.0)  1,853 (21.2)  1,197 (13.7)  764 (8.74) |  |
| 2018 | -Total adult population*, n (%)  Male  Female  -Age distribution (years), n (%)  20-29  30-39  40-49  50-59  60-69  ≥ 70 | 11,983,418 (100.0)  5,773,286 (48.2)  6,210,132 (51.8)  2,174,948 (18.1)  1,917,696 (16.0)  2,559,884 (21.4)  2,418,305 (20.2)  1,676,492 (14.0)  1,236,093 (10.3) | 1,065,435 (100.0)  507,303 (47.6)  558,132 (52.4)  209,888 (19.7)  169,937 (16.0)  216,134 (20.3)  213,737 (20.1)  149,162 (14.0)  106,577 (10.0) | 9,112 (100.0)  4,386 (48.1)  4,726 (51.9)  1,700 (18.7)  1,459 (16.0)  1,914 (21.0)  1,929 (21.2)  1,242 (13.6)  868 (9.53) |  |
| 2019 | -Total adult population*, n (%)  Male  Female  -Age distribution (years), n (%)  20-29  30-39  40-49  50-59  60-69  ≥ 70 | 11,717,075 (100.0)  5,648,091 (48.2)  6,068,984 (51.8)  2,047,349 (17.5)  1,801,993 (15.4)  2,428,135 (20.7)  2,445,216 (20.9)  1,708,039 (14.6)  1,286,343 (11.0) | 995,253 (100.0)  477,472 (48.0)  517,781 (52.0)  169,484 (17.0)  155,012 (15.6)  200,406 (20.1)  211,407 (21.2)  149,478 (15.0)  109,466 (11.0) | 8,790 (100.0)  4,215 (48.0)  4,575 (52.0)  1,646 (18.7)  1,270 (14.4)  1,764 (20.1)  1,916 (21.8)  1,306 (14.9)  888 (10.1) |  |

* Including adult persons whose age ≥ 20 years, ** including adult persons whose age ≥ 18 years

**Supplementary Figures**

**Figure S1: The histograms of eGFR levels in the participants stratified by sex and age groups (in terms of frequency and percentage).**

**Figure S2: Correlations of eGFR and age in males and females**.


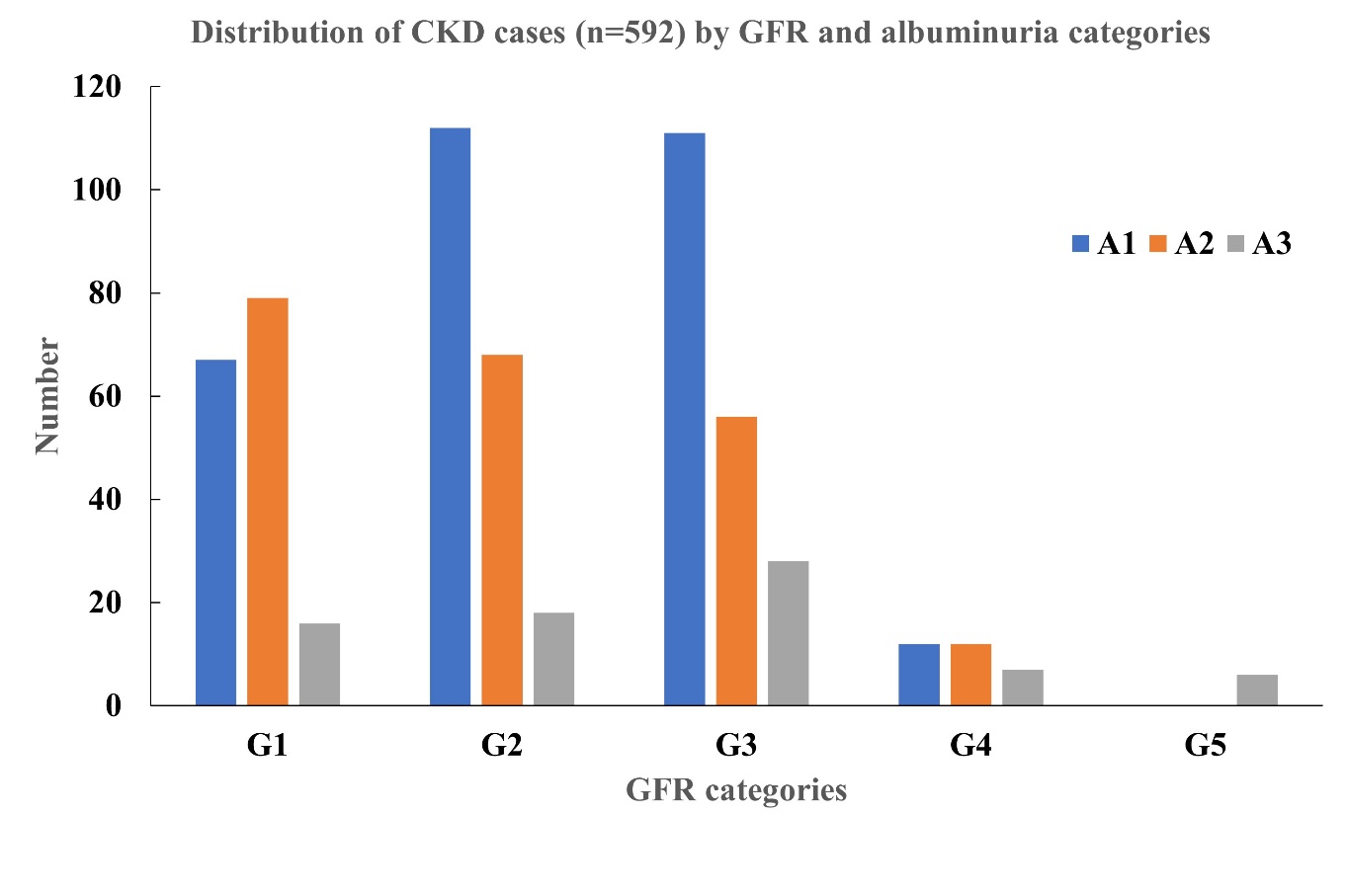


**Figure S3: Proportions of albuminuria categories in each CKD stage**


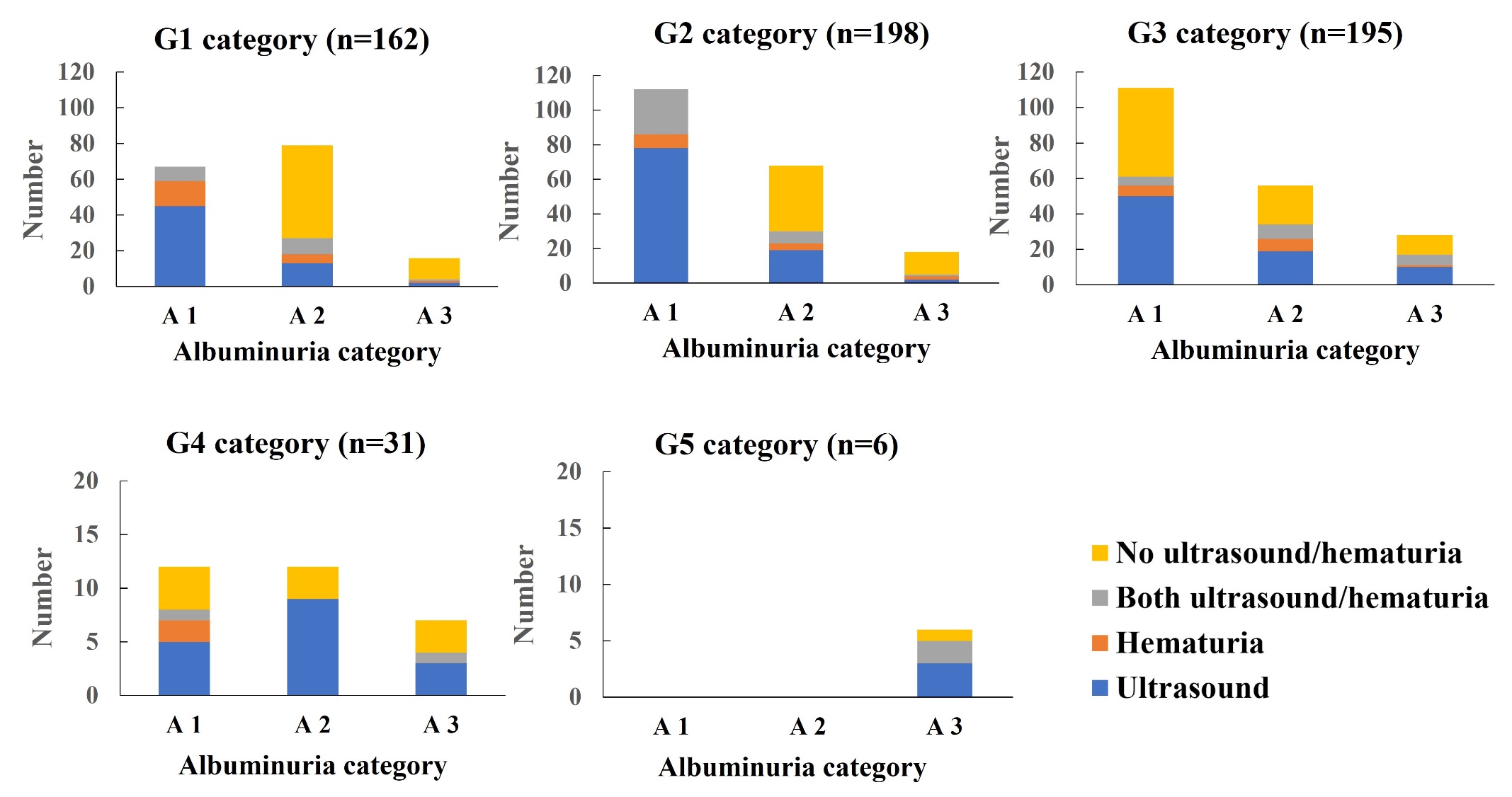


**Figure S4: Distributions of CKD cases in each albuminuria category which CKD was defined by hematuria and/or ultrasound criteria**


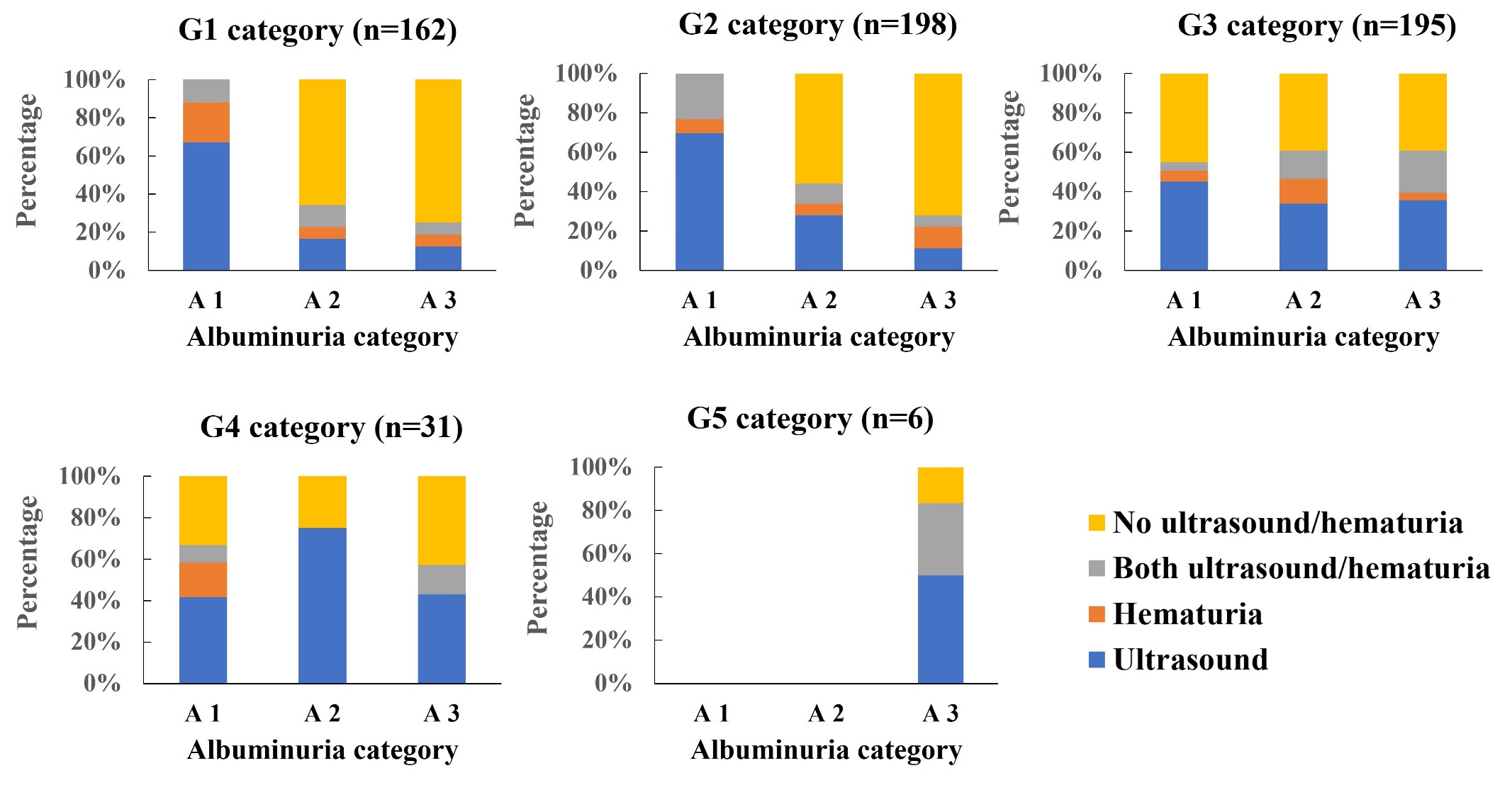


**Figure S4 (continued): Distributions of CKD percentage in each albuminuria category which CKD was defined by hematuria and/or ultrasound criteria**
